# Elucidating the role of 4-hydroxy-2(3H)-benzoxazolone in chronic alcoholic liver disease via transcriptomics and metabolomics

**DOI:** 10.3389/fphar.2024.1447560

**Published:** 2024-09-11

**Authors:** Jun-Fei Lu, Shang-Ping Xing, Xia Wei, Chun-Xia Yang, Gen-Shi Zhao, Xiao-Lin Ma, Xue-Mei Sun, Hong-Wei Guo, Zhi-Heng Su, Bin Fang, Jun Lin, Yan-Ying Liu, Dan Zhu

**Affiliations:** ^1^ Pharmaceutical College, Guangxi Medical University, Nanning, China; ^2^ Department of Pharmacy, College and Hospital of Stomatology, Guangxi Medical University, Nanning, China; ^3^ Key Laboratory of Longevity and Geriatric Diseases, Ministry of Education, Guangxi Medical University, Nanning, China; ^4^ Guangxi Key Laboratory for Bioactive Molecules Research and Evaluation, Nanning, China; ^5^ Guangxi Beibu Gulf Marine Biomedicine Precision Development and High-value Utilization Engineering Research Center, Nanning, China; ^6^ Guangxi Health Commission Key Laboratory of Basic Research on Antigeriatric Drugs, Nanning, China

**Keywords:** chronic alcoholic liver disease, 4-hydroxy-2(3H)-benzoxazolone, oxidative stress, immune inflammation, transcriptomics, metabolomics, NF-κB, glycerophospholipid metabolism

## Abstract

**Background:**

Chronic alcoholic liver disease (CALD) is a global health problem which includes multiple pathological processes such as immune inflammation and oxidative stress. 4-hydroxy-2(3H)-benzoxazolone (HBOA), an alkaloid isolated from *Acanthus ilicifolius* L, has been shown to exert hepatoprotective and immunomodulatory effects. However, its effects on CALD remain unclear. This study aimed to investigate the effects and underlying mechanisms of HBOA on CALD.

**Methods:**

Rats were administered alcohol by gavage continuously for 12 weeks to establish the CALD model, and then treated with HBOA by gavage for 4 weeks. Transcriptomics and metabolomics were used to predict the potential mechanisms of the effects of HBOA on CALD. Liver histology and function, oxidative stress, inflammatory cytokines, and the TLR4/NF-κB pathway components were evaluated.

**Results:**

HBOA significantly improved alcohol-induced liver injury and steatosis. It decreased the expression levels of pro-inflammatory cytokines (tumour necrosis factor-α [TNF-α], interleukin (IL)-1β, and IL-6), and increased the activities of antioxidant enzymes (superoxide dismutase [SOD], glutathione [GSH], and glutathione peroxidase [GSH-Px]). Western blotting confirmed that HBOA treatment largely diminished NF-κBp65 nuclear translocation. Comprehensive transcriptomics and metabolomics analyses indicated that HBOA regulated the glycerophospholipid metabolism pathway to achieve therapeutic effects in rats with CALD.

**Conclusion:**

HBOA has a therapeutic effect on rats with CALD. Its mechanism of action mainly affects the glycerophospholipid metabolic pathway to promote lipid metabolism homeostasis by regulating the expression of *Etnppl*, *Gpcpd1,* and *Pla2g4c*. In addition, it may also inhibit the TLR4/NF-κB signaling pathway, thereby reducing the immune-inflammatory response.

## 1 Introduction

The liver has metabolic, detoxification, and immune functions (Trefts et al., 2017). Alcohol is mainly metabolized by the liver, and chronic alcoholic liver disease (CALD) caused by alcohol abuse contributes significantly to the global mortality and disease burden of liver-related deaths (Aslam and Kwo, 2022); moreover, there are no satisfactory medical treatments for CALD. The pathogenesis of CALD is often characterized by steatosis, immunoinflammatory, and oxidative stress, and is closely associated with multiple metabolic alterations (Takeuchi et al., 2021). Currently, clinical treatment for patients with chronic alcoholic liver disease is mainly based on immunomodulation, hepatoprotection, and complication control programs, but some patients still have poor results.

Long-term sustained alcohol stimulation activates the TLR4/NF-κB transduction pathway and promotes the development of CALD (Tang et al., 2023). TLR4 directly recognizes and binds pathogen-associated molecular patterns, triggering downstream signaling that leads to the release of inflammatory cytokines and chemokines (Sahlman et al., 2016), while transcription of pro-inflammatory factors such as IL-6, IL-1β, and TNF-α and inflammation-related genes are mainly regulated by NF-κB activation (Bieghs and Trautwein, 2013). The inhibition of the TLR4/NF-κB signaling pathway has been shown to reduce alcohol-induced liver damage and inflammatory responses (Liu et al., 2022). The accumulation of acetaldehyde and free radicals in the liver during the metabolism of alcohol causes oxidative stress damage to the liver, which exacerbates pathological changes such as steatosis, inflammatory cell infiltration, and liver enlargement (Li et al., 2014). Thus, the NF-κB signaling pathway may regulate immunoinflammatory response and oxidative stress in CALD.

Chronic alcohol abuse may cause disturbances in the body’s lipid metabolic homeostasis (You and Arteel, 2019). Lipids are involved in the composition of cell membranes, subcellular membranes, and lipoproteins, and are also important in cell signaling, maintenance of homeostasis, inflammation, and immune response (Buechler and Aslanidis, 2020). When lipid accumulation or lipotoxicity occurs in the body, it can damage the cell membrane structure and thus aggravate liver damage (Zámbó et al., 2013). Glycerophospholipid is closely related to alcoholic liver disease (Cao et al., 2022), and its homeostasis imbalance can cause adverse consequences such as liver damage and steatohepatitis (Wang et al., 2021). Modulation of lipid homeostasis may be an effective strategy for the treating CALD.

**FIGURE 1:**
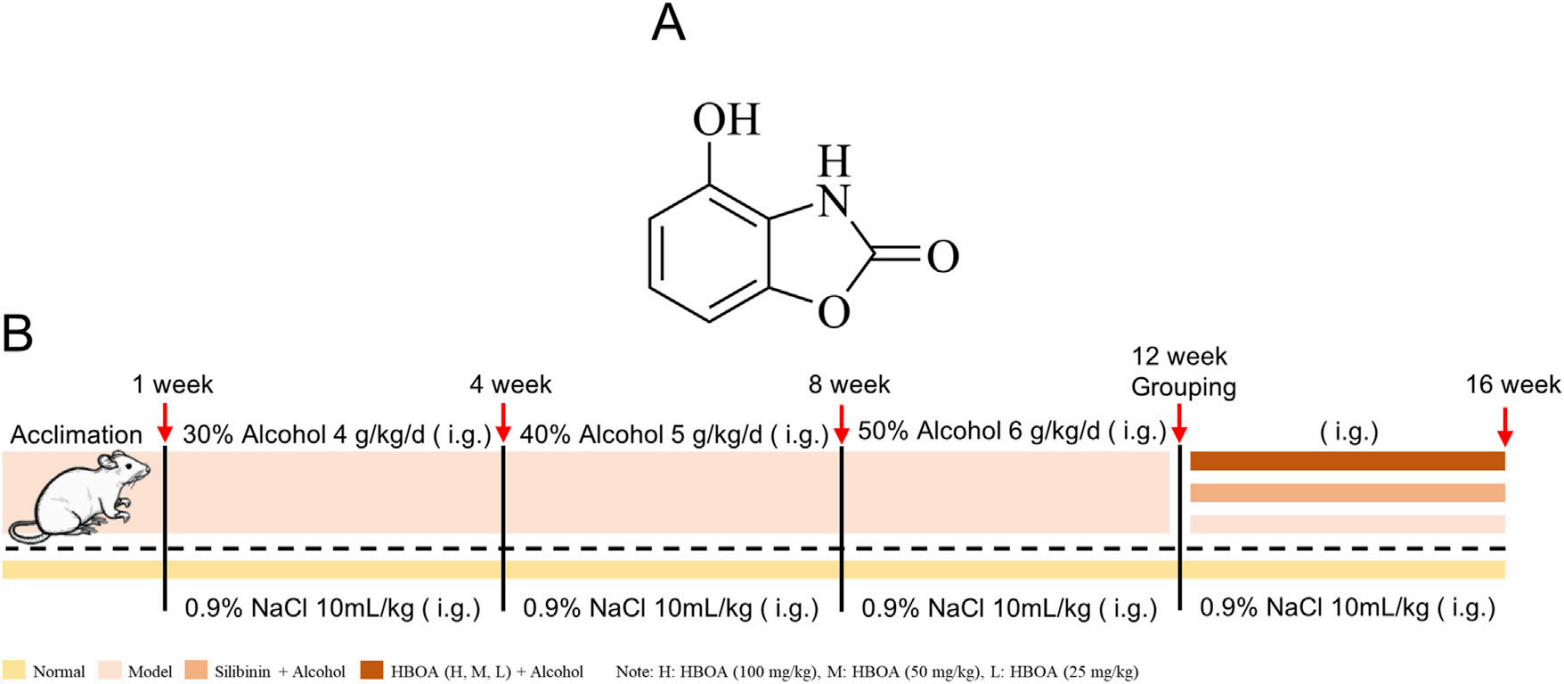
**(A)** Chemical formula of 4-hydroxy-2(3H)-benzoxazolone (HBOA). **(B)** Modeling method of chronic alcoholic liver disease (CALD).

*Acanthus ilicifolius* L. is an erect shrub found in coastal areas and is believed to be effective in treating heat toxins, carbuncles, boils, and enlarged liver and spleen in Chinese folk (Liu et al., 2013). The alcoholic extract of *Acanthus ilicifolius* has excellent hepatoprotective (Babu et al., 2001) and antitumor (Babu et al., 2002) effects. 4-hydroxy-2(3H)-benzoxazolone (HBOA) (Figure 1A) is one of the main components isolated from *A. ilicifolius*. In our previous study, HBOA was synthesized and found to have a strong hepatoprotective effect (Liu et al., 2013; Sun et al., 2019). Previous studies also found that it also inhibited the immune-inflammatory response in serum-induced immune liver injury, suggesting that it may have immunomodulatory effects. To comprehensively understand the therapeutic effects of HBOA in various liver diseases and develop it as a promising agent for the treatment of liver injury, multiple experimental models of liver disease need to be investigated. The effects of HBOA on CALD remain unclear; therefore, this study focused on the hepatoprotective effects and underlying mechanisms of HBOA on CALD using transcriptomic and metabolomic technology to elucidate theoretical evidence for the application of HBOA in the treatment of CALD.

## 2 Materials and methods

### 2.1 Animals and treatment

A total of 48 Sprague Dawley male rats weighing 200 ± 10 g were purchased from the Experimental Animal Center of Guangxi Medical University (Guangxi, China). The care and treatment of the animals was conducted following the guidelines established by the Public Health Service Policy on the Humane Care and Use of Laboratory Animals. All the procedures of animal care and experimentation were approved by the Animal Ethics Committee of Guangxi Medical University, Guangxi, China, approval number: (202106003). The experimental schedule is shown in Figure 1B. In brief, rats were randomly divided into 6 groups (n = 8): the normal group, model group, Silibinin (26.25 mg/kg) group and, HBOA groups (100, 50, 25 mg/kg) after 7 days of acclimation. The rats in the last five groups were used to establish a CALD model by intragastric administration of edible alcohol (Beijing Red Star Co. Ltd., Beijing, China), while the normal group was given basal feed for 12 weeks. After successful modeling, the rats were subjected to various drugs except for the normal and model groups for 4 weeks, and the animals in the normal and model groups were given an equal amount of 0.9% saline. Silibinin and HBOA were administered by gavage once daily. In this study, Silibinin (Tianjin Tasly Shengte Pharmaceutical Co., Ltd., Tianjin, China), natural medicinal treatment for liver protection, was selected as the positive control, and the therapeutic doses of Silibinin and HBOA [purity >98%, Pharmaceutical Chemistry Laboratory, Guangxi Medical University, Guangxi, China (Wang et al., 2020)] were selected based on our previous study (Sun et al., 2019; Wang et al., 2018). The alcohol concentrations used in the induction and maintenance of the disease model were as follows: 30% alcohol, 4.0 g/kg/d from 1 to 4 weeks; 40% alcohol, 5.0 g/kg/d from 5 to 8 weeks; 50% alcohol, 6.0 g/kg/d from 9 to 12 weeks; and 50% alcohol, 6.0 g/kg/d from 13 to 16 weeks. During the treatment period, staggered gavage of edible alcohol was used to maintain the model, in addition to the administration of the corresponding medication. At the end of the treatment, the rats were anaesthetized using 20% urethane (1 g/kg), and their livers and serum were extracted for further experimental procedures.

### 2.2 Observation of the general status of rats and changes in the liver index

The diet, hair, activity, and growth of rats were observed during the experiment. The liver index was calculated based on the body weight and liver mass: liver index = (liver wet weight/body mass) × 100%.

### 2.3 Biochemical analysis

Serum levels of alanine aminotransferase (ALT), aspartate aminotransferase (AST), and alkaline phosphatase (ALP) were detected using the corresponding biochemical assay kits (Nanjing Jiancheng Bioengineering Institute, Nanjing, China). Total cholesterol (TC), total triglyceride (TG), high-density lipoprotein cholesterol (HDL-C), and low-density lipoprotein cholesterol (LDL-C) in the serum were determined using BS-2000 automatic biochemical analyzer (Shenzhen Mindray Bio-Medical Electronics Co., Ltd., Shenzhen, China).

### 2.4 Pathological examination

Hepatic histological changes were observed by Hematoxylin-eosin (H&E) staining. Briefly, liver tissues were fixed with 4% paraformaldehyde for 48 h, after which these were embedded with paraffin wax and cut into 4 μm sections. H&E staining of tissue sections was performed according to standard procedures.

Steatosis was observed by Oil red O staining. Briefly, a portion of fresh rat liver tissue was taken and cryosectioned. Sections were stained with oil red O according to standard procedures.

### 2.5 Oxidative stress and lipid peroxidation levels

The activities of superoxide dismutase (SOD), glutathione (GSH), malondialdehyde (MDA) in liver tissue homogenates and glutathione peroxidase (GSH-Px) in serum were assayed according to the manufacturer’s instructions of the SOD, GSH, MDA and, GSH-Px Assay Kit (Nanjing Jiancheng Bioengineering Institute, Nanjing, Jiangsu, China).

### 2.6 Determination of inflammatory cytokines in serum

Inflammatory cytokines in liver tissue, including IL-6, IL-1β, TNF-α, as well as lipopolysaccharides (LPS), were determined using the corresponding commercial enzyme-linked immunosorbent assay (ELISA) kits (Shanghai Vancove Biotechnology Co., Ltd., Shanghai, China) according to the manufacturer’s instructions.

### 2.7 Transcriptome sequencing

Liver tissues of 3 rats (Approx. 100 mg each) in each group were randomly selected from the normal group, model group and HBOA (100 mg/kg) group for RNA sequencing. The RNA sequencing was performed by Shanghai Personal Biotechnology Cp. Ltd., Shanghai, China. Total RNA was isolated using Trizol Reagent (Invitrogen Life Technologies, Carlsbad, United States). RNA sequencing libraries were constructed using the NEBNext Ultra II RNA Library Prep Kit for Illumina. Synthesis of cDNA was carried out after enrichment of mRNA using Oligo (dT) magnetic beads. cDNA fragments of approximately 400–500 bp size were screened by polymerase chain reaction using AMPure XP beads. These were then amplified and purified to obtain the final libraries. The libraries were tested for quality and concentration, and the multiplexed DNA libraries were homogenized and mixed in equal volumes. The mixed libraries were gradually diluted, quantified, and then sequenced in PE150 mode on the NovaSeq 6,000 platform (Illumina). Differential expression analysis between groups was performed using DESeq (1.39.0). Differential expression fold |log2FoldChange| >1 and P-value <0.05 were used as conditions to screen differentially expressed genes for the next analysis. The differentially expressed genes were screened for subsequent gene ontology (GO) and Kyoto Encyclopedia of Genes and Genomes (KEGG) enrichment analyses.

### 2.8 Molecular docking

The uniport database (https://www.uniprot.org/) was used to retrieve receptor proteins MAPK, FoXO, AMPK, JAK, STAT, NF-κB, PPAR, Ras, and TLR4 (Uniprot ID: P28482, Q12778, P54646, P23458, P40763, P19838, Q07869, P20936, and O00206, respectively). Water and ligands were removed using PyMOL version 2.3.4. The PubChem database (https://pubchem.ncbi.nlm.nih.gov) was used to obtain the spatial data file structure file of HBOA (CAS: 28955-70-6), and AutoDockTools version 1.5.6 was used for hydrogenation, electronation, and other operations. AutoDock and PyMOL were used for molecular docking and data visualization, respectively.

### 2.9 Metabolomics analysis

Metabolomics studies were performed on liver tissues (Approx. 100 mg each) of 8 rats from each of the normal, model and HBOA (100 mg/kg) groups. These liver tissues were used to prepare separate liver homogenates using pre-cooled methanol (800 μL/sample), placed on ice for 15 min, and then centrifuged (12,000 rpm, 10 min, 4°C). A 400 μL aliquot of the supernatant from each group was filtered using a 0.22 μm microporous filter membrane. 10 μL of each of the above samples were mixed and used as quality control (QC) samples. The supernatant was analyzed by Ultra-performance liquid chromatography equipped with quadrupole time-of-flight mass spectroscopy (UPLC-Xevo G2-XS QTof, Waters Corp., United States). Chromatographic separations were performed using an Acquity UPLC^®^ BEH C18 column (50 mm × 2.1 mm, 1.7 μm, Waters Corp.) with the column and autosampler tray temperatures maintained at 45°C and 4°C, respectively. The composition of the mobile phase A (water at 0.1% formic acid) and B (acetonitrile-0.1% formic acid) at a flow rate of 0.3 mL/min. The elution gradient is presented in Table 1. Each injection volume was 5 µL, injected over 15 min. Mass spectrometry was performed in positive ion mode in the mass range of 50–1,200 Da. Metabolomics data were analyzed using SIMCA^@^ version 14.1, Masslynx version 4.1, Progenesis QI version 2.4, and EZinfo version 3.0 (Waters Inc.). The differential metabolites obtained by screening were used for subsequent analysis using RStudio version 2022.07.2 with the various packages.

**TABLE 1:** Sample solvent gradient elution procedure.

Time (min)	Flow velocity (mL/min)	A%	B%
0	0.3	95	5
1	0.3	80	20
2.5	0.3	60	40
9	0.3	10	90
10	0.3	0	100
12.5	0.3	0	100
14	0.3	95	5

### 2.10 Combined transcriptome and metabolome analyses

To explore the relationship between differentially expressed genes and metabolites, correlation analysis of transcriptomics and metabolomics data was performed using RStudio with various packages. Cluster heatmap correlation analysis was performed, and nine quadrants were plotted using the R packages. Joint-Pathway analysis was performed using the website (MetaboAnalyst 5.0).

### 2.11 Western blotting

Rat liver tissues were removed from −80°C storage, cleaned, and added to the premix (radioimmunoprecipitation assay buffer: protease inhibitor: phosphatase inhibitor, 100:1:1) for pulverization and homogenization. The resulting slurry was lysed on ice for 20 min and then centrifuged (12,000 rpm, 4°C, 15 min). The supernatant was collected, and the total protein content was determined according to the manufacturer’s instructions of the bicinchoninic acid protein assay kit (Beyotime Biotechnology Co., Ltd., Shanghai, China). A protein loading buffer was added to the supernatant, after which it was boiled (15 min) and denatured. Protein electrophoresis was performed using sodium dodecyl sulfate-polyacrylamide gels; the separated proteins were transferred to polyvinylidene fluoride membranes and the membrane strips were shaken for 10 min in a blocking buffer. The membrane was washed with Tris-buffered saline Tween-20, and then incubated overnight at 4°C with the corresponding primary antibody: TLR4 (Proteintech, China), MyD88 (BOSTER, United States), p-IKKα/β (Cell Signaling, United States), IKKα/β (Bioss, China), p-IκBα (Cell Signaling, United States), IκBα (Proteintech, China), p-NF-κBp65 (Affinify, United States), NF-κBp65 (BOSTER, United States), Lamin B1 (Proteintech, China), GAPDH (Abmart, China). Subsequently, the membranes were removed and washed and then incubated in a secondary antibody for 30 min to 1 h with flat shaking under light-proof conditions. Finally, the bands were detected by Image Studio Lite software (LI-COR Biosciences, NE, United States), and the strips were quantified by ImageJ 2x version 2.1.4.6 ud4.

### 2.12 Statistical analyses

Statistical analysis was performed using SPSS version 17.0 for Windows. Differences between the groups were assessed using a one-way analysis of variance with Tukey’s test for *post hoc* multiple comparisons. The data were presented as means ± standard deviation. A P-value < 0.05 was considered statistically significant.

## 3 Results

### 3.1 HBOA ameliorates liver tissue injury and steatosis in rats with CALD

**FIGURE 2:**
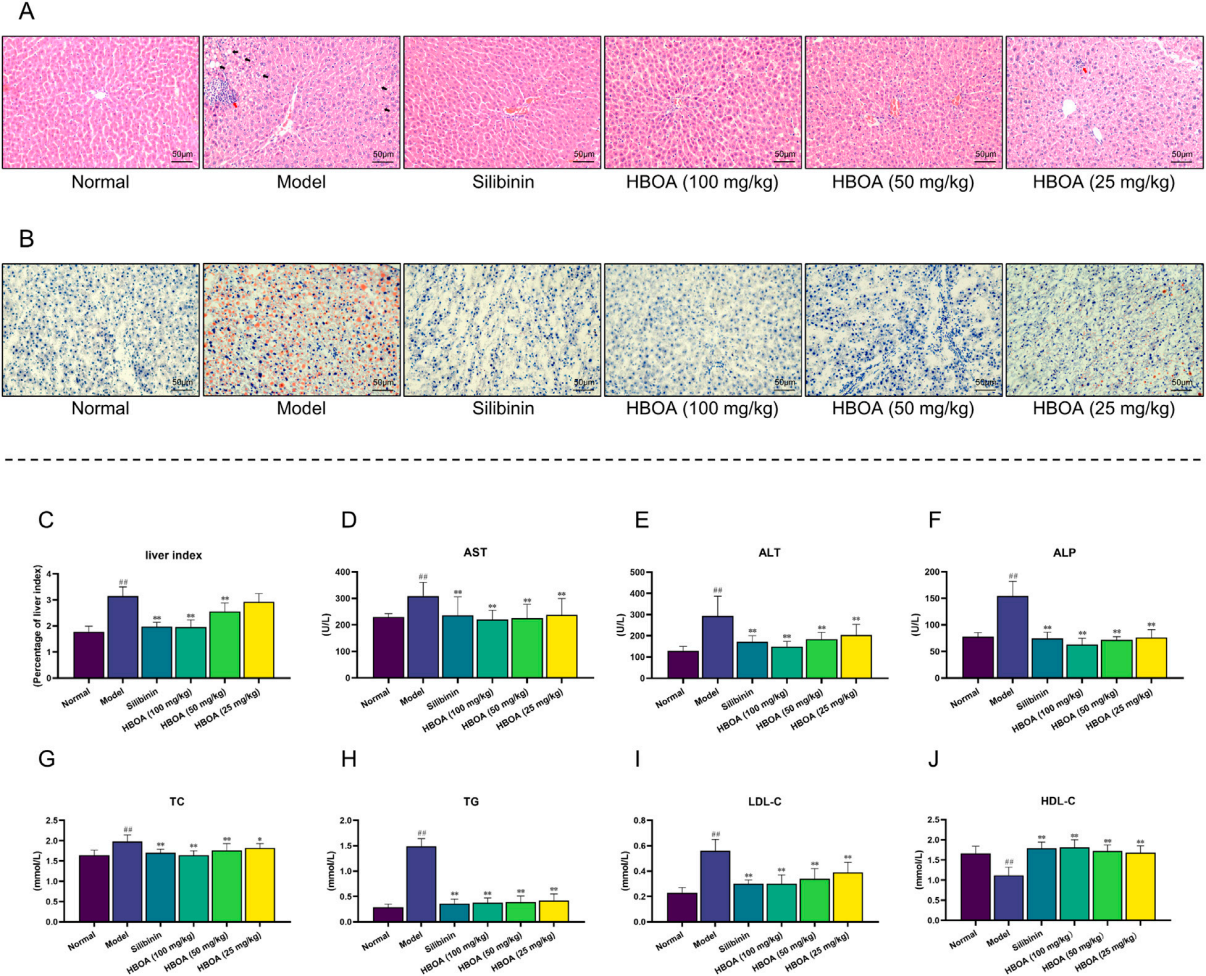
HBOA treatment ameliorates CALD. **(A)** Hepatic histological changes were observed by H&E staining. **(B)** Steatosis was observed by Oil red O staining. **(C–F)** Results of the biochemical analysis. **(C)** Liver index, **(D)** AST, **(E)** ALT, and **(F)** ALP of CALD rats. The content of **(G)** TC, **(H)** TG, **(I)** LDL-C, and **(J)** HDL-C in serum was detected using an automatic biochemical analyzer. ^*^ *P* < 0.05, ^**^ *P* < 0.01 versus the model group; ^##^ *P* < 0.01 versus the normal group.

To evaluate the protective effects of HBOA on rats with CALD, we first examined the histological changes in rat liver tissues by H&E staining and Oil Red O red staining. As shown in Figure 2A, live tissues of the CALD model group exhibited obvious liver tissue lesions, as evidenced by the formation of hepatocytes with lightly stained and pale cytoplasm, poorly defined hepatocyte boundaries, and the presence of inflammatory infiltrates (Red arrows) and marked fatty vacuoles around the liver lobules (Black arrows). In contrast, the groups treated with HBOA or Silibinin showed a significant reduction in the above-mentioned liver lesions compared with the model group. Among the HBOA treatment groups, the therapeutic effect was dose-dependent. The Oil Red O staining pathology in the CALD model group showed extensive red staining and diffuse red lipid droplet distribution, while the HBOA and Silibinin groups showed alleviation of these abnormal changes compared with the model group (Figure 2B). These results suggest that treatment with HBOA significantly improved liver damage and steatosis in rats.

As shown in Figures 2C–J, biochemical analyses indicated that the levels of the liver index, AST, ALT, ALP, TC, TG, and LDL-C for the CALD model group were markedly increased, and that of HDL-C was dramatically reduced compared with the levels for the normal group (*P* < 0.01). HBOA and Silibinin treatment notably reversed these abnormal changes (*P* < 0.05). HBOA dose-dependently decreased the level of liver index, ALT, ALP, TC, and LDL-C while it increased that of HDL-C. As indicated here, the biochemical results were in accord with the histological changes observed, indicating that HBOA improved liver function and lipid profile in rats with CALD.

### 3.2 HBOA attenuates inflammation and oxidant stress

**FIGURE 3:**
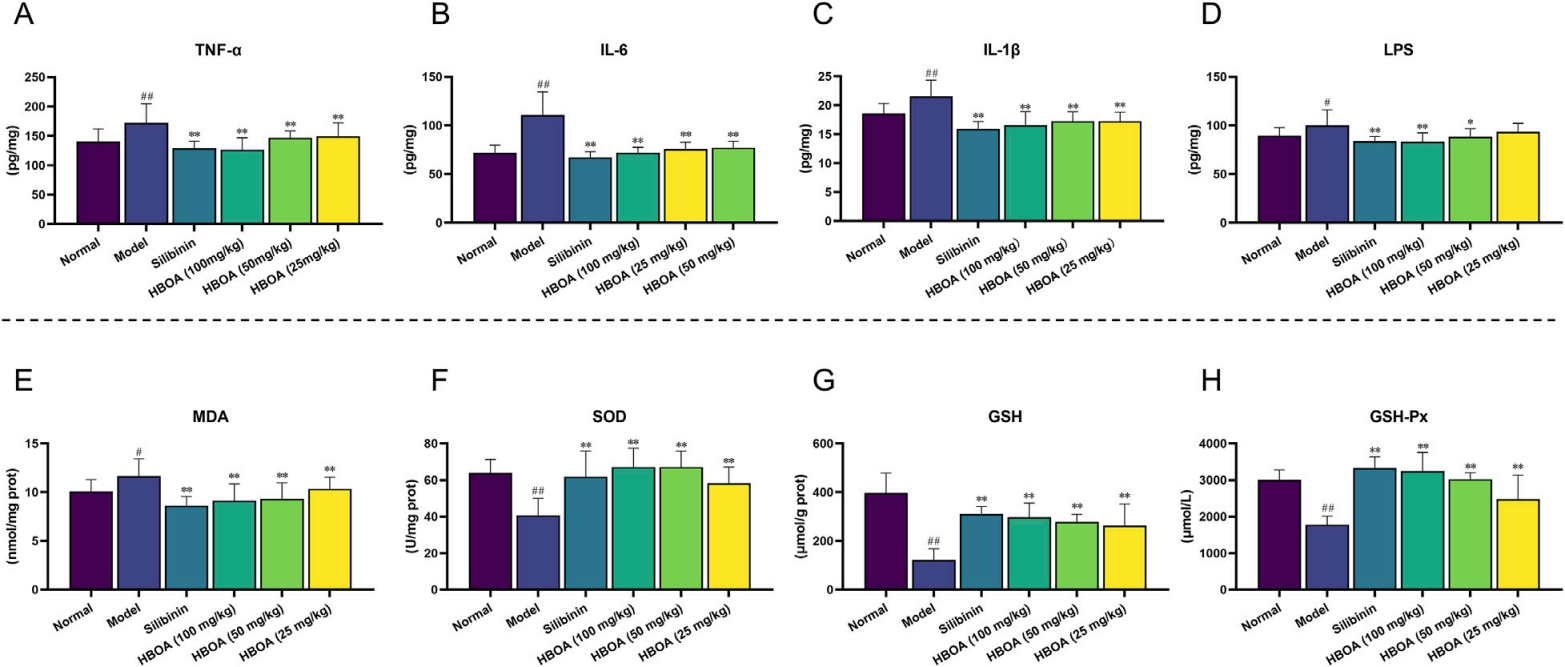
HBOA treatment attenuated inflammation and oxidant stress in rats with CALD. **(A–C)** The content of the proinflammatory cytokines. **(A)** TNF-α, **(B)** IL-6, **(C)** IL-1β, and **(D)** LPS in liver tissue were measured by ELISA kits. The content of **(E)** MDA, **(F)** SOD, **(G)** GSH, and **(H)** GSH-Px was detected by commercial kits. ^**^ *P* < 0.01 versus the model group; ^##^ *P* < 0.01 versus the normal group.

To verify whether HBOA relieved CALD by inhibiting inflammatory responses, the levels of pro-inflammatory cytokines were measured using ELISA kits. As shown in Figures 3A–C, the levels of TNF-α, IL-1β, and IL-6 were markedly increased for the CALD model group compared with the normal group (*P* < 0.01). The expression of TNF-α, IL-1β, and IL-6 in rats with CALD treated with HBOA (25, 50, or 100 mg/kg) gradually decreased as compared with that observed for the CALD model group (*P* < 0.01). Additionally, LPS was significantly increased for the CALD model group, and was positively correlated with inflammation (Figure 3D); however, it was markedly decreased after HBOA treatment (*P* < 0.05).

We further assessed the concentration of MDA and the activity of antioxidant enzymes, which play crucial roles in oxidative stress responses. As shown in Figures 3E–H, MDA activity was significantly increased, and the activities of SOD, GSH, and GSH-Px were markedly decreased for the CALD model group (*P* < 0.01). Treatment with HBOA effectively attenuated the abnormal increase of MDA and enhanced the activities of SOD, GSH, and GSH-Px (*P* < 0.01). These results indicate that HBOA reduces the inflammatory response and oxidative stress in rats with CALD.

### 3.3 Transcriptome analysis

**FIGURE 4:**
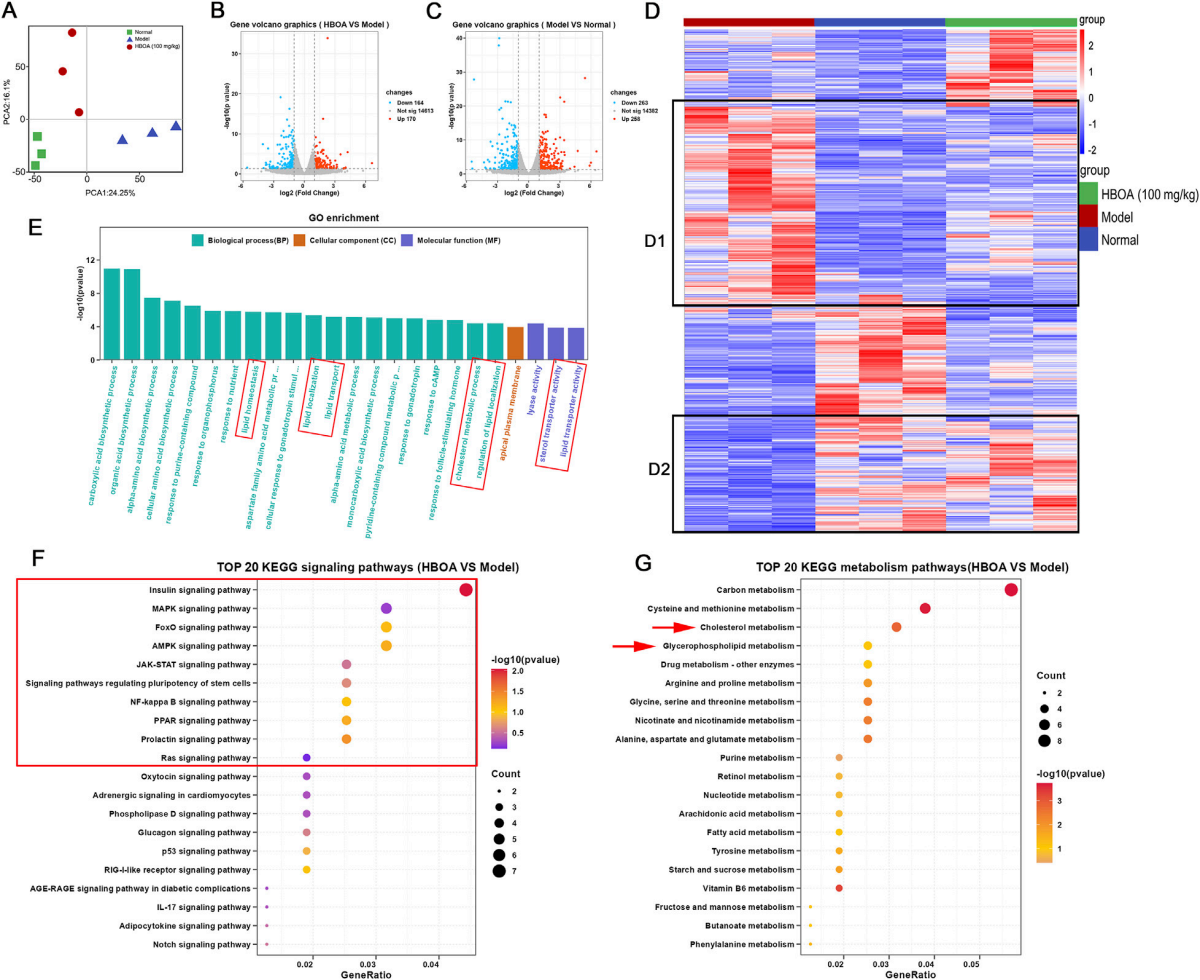
HBOA-induced transcriptomic alterations. **(A)** PCA diagram. **(B)** Volcano plot (HBOA vs. Model). **(C)** Volcano plot (Model vs. Normal). **(D)** Heatmap diagram (red plots indicate a high level of gene expression). **(E)** Gene Ontology (GO) analysis; **(F)** KEGG signaling pathway enrichment (HBOA vs. model). **(G)** KEGG metabolic pathway enrichment (HBOA vs. model).

Transcriptomic techniques were used to predict the potential targets and associated regulatory pathways for rats with CALD treated with HBOA. Principal component analysis (PCA) plots (Figure 4A) showed a discrete distribution among the normal, model, and HBOA (100 mg/kg) groups, indicating significant differences in genes among these groups. In addition, the heatmap (Figure 4D) showed that the expression of some genes was significantly increased or decreased as a result of long-term alcohol intervention and that HBOA treatment reversed these aberrantly expressed genes. The volcano in Figures 4B, C showed that 258 genes were upregulated, and 263 genes were downregulated for the CALD model group compared with those for the normal group and that 170 genes were upregulated and 164 genes were downregulated after HBOA intervention as compared to those for the CALD model group.

The results of GO enrichment analysis are indicated in Figure 4E and show that the affected biological processes were mainly related to lipid homeostasis, localization, and transport, while the affected molecular functions were mainly related to the transporter activities of lipids and cholesterol. KEGG enrichment analysis showed that the differential genes were enriched in multiple signaling and metabolic pathways. To further understand the affected metabolic and signaling pathways, we performed KEGG analysis for metabolic and signaling pathways separately. As indicated by the KEGG signaling pathway enrichment analysis shown in Figure 4F, HBOA may attenuate CALD by affecting the MAPK, FoxO, AMPK, JAK-STAT, NF-kappa B, PPAR, and Ras signaling pathways. As shown in Figure 4G, KEGG metabolic pathway enrichment analysis indicated that HBOA may attenuate CALD by affecting cholesterol and glycerophospholipid metabolism, which is consistent with the findings of the GO analysis.

### 3.4 Molecular docking

**FIGURE 5:**
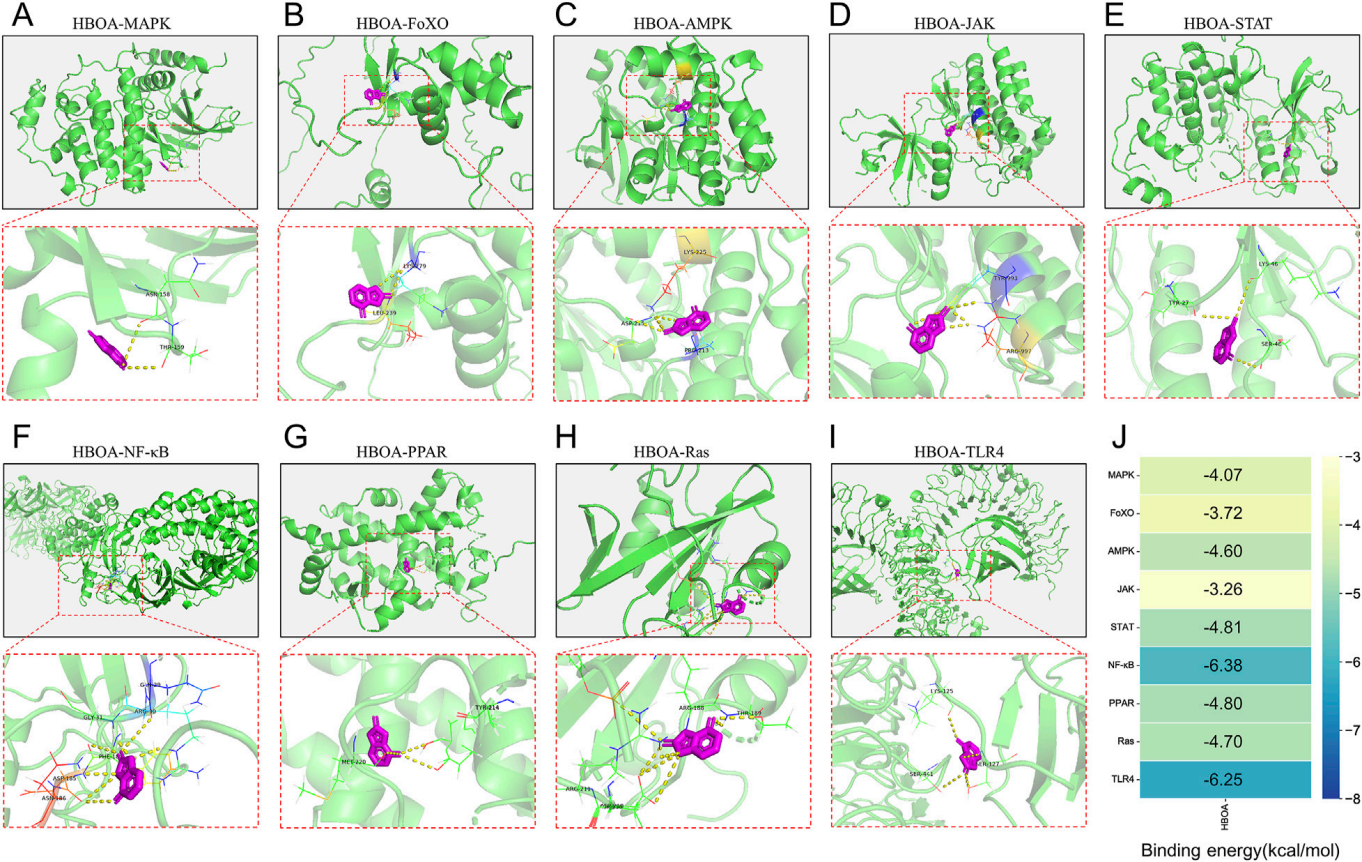
Molecular docking diagram. **(A)** HBOA-MAPK **(B)** HBOA-FoXO, **(C)** HBOA-AMPK, **(D)** HBOA-JAK, **(E)** HBOA-STAT, **(F)** HBOA-NF-κB, **(G)** HBOA-PPAR, **(H)** HBOA-Ras, and **(I)** HBOA-TLR4. **(J)** Binding energy score diagram obtained by molecular docking.

Molecular docking was performed to screen for targets that are most likely to have a therapeutic effect by binding to HBOA and generating activity. This was done to predict the protein-binding activities of the top 10 key proteins with HBOA of the signaling pathway enriched by KEGG. As shown in Figure 5 and Table 2, MAPK, FoxO, AMPK, JAK, STAT, NF-κB, PPAR, and Ras proteins could all bind to HBOA, with NF-κB exhibiting the lowest binding energy (−6.38 kcal/mol), and the highest binding activity with HBOA. Further molecular docking of TLR4, an upstream target of NF-κB and associated with inflammation onset, revealed that its binding energy was also less than −6 kcal/mol, suggesting that the TLR4/NF-κB signaling pathway may be a target pathway for HBOA treatment in rats with CALD.

**TABLE 2:** Binding energy and bound amino acid residues obtained by docking HBOA with key protein molecules of the top 10 signaling pathway enriched by transcriptomic KEGG.

Proteins	Uniprot ID	Binding energy/(kcal/mol)	Amino acid residues of HBOA bound to proteins to form hydrogen bonds
MAPK	P28482	−4.07	ASN-158, THR-159
FoXO	Q12778	−3.72	LEU-239, LYS179
AMPK	P54646	−4.60	LYS225, ASP215, PRC213
JAK	P23458	−3.26	TYR-993, ARG-997
STAT	P40763	−4.81	LYS-46, TYR-27, SER-4ε
NF-κB	P19838	−6.38	GLN-29, ARG-30, GLY-31, PHE-184, ASP-185, ASN-186
PPAR	Q07869	−4.80	MET-220, TYR-214
Ras	P20936	−4.70	THR-189, ARG-188, ARG-211, ASP-210
TLR4	O00206	−6.25	LYS-125, SER-127, SER-441

### 3.5 HBOA inhibits the TLR4/NF-κB signaling pathway

**FIGURE 6:**
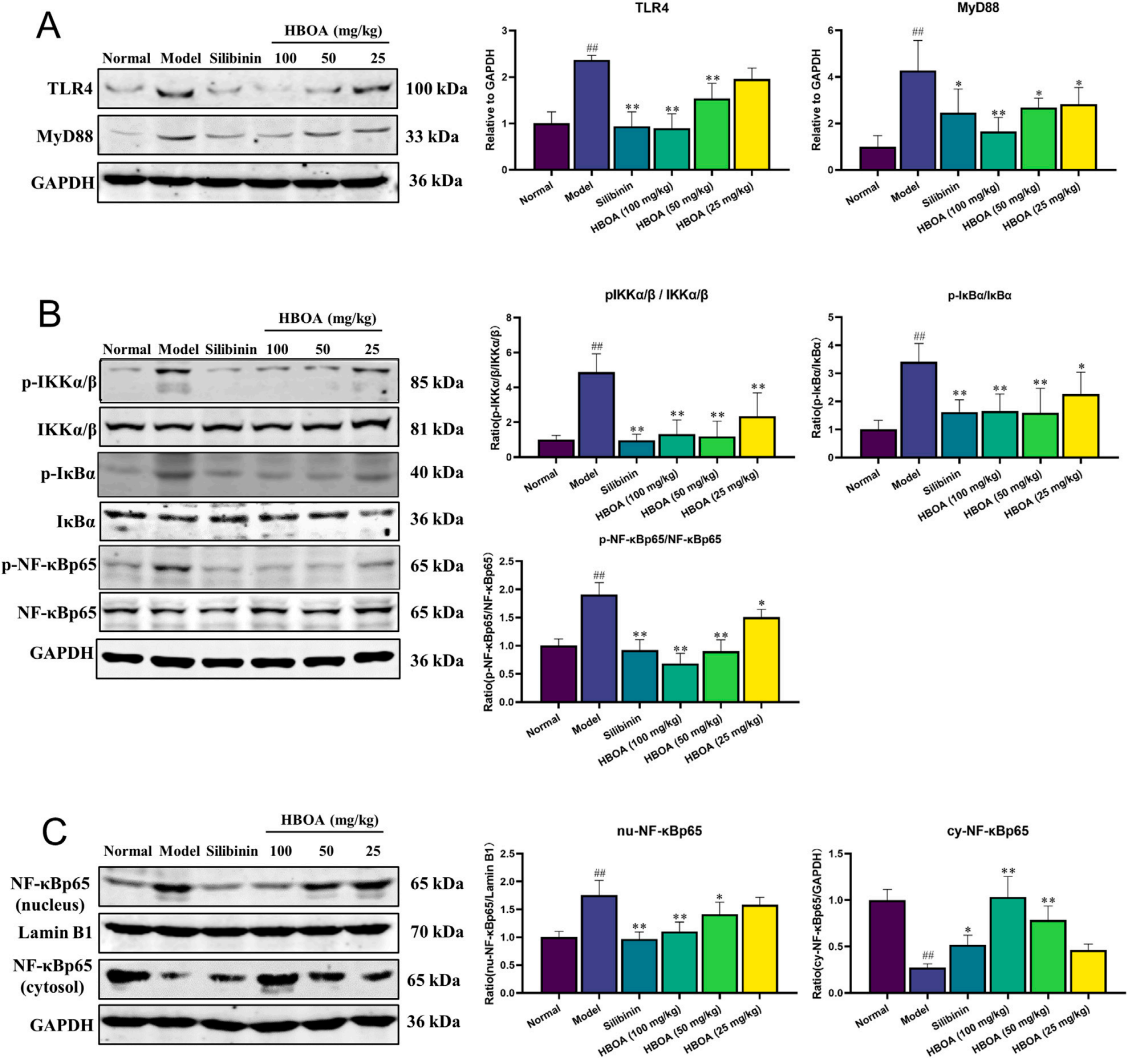
HBOA inhibits the TLR4/NF-κB signaling pathway. The protein expressions of **(A)** TLR4 and MyD88, **(B)** p-IKKα/β, IKKα/β, p-IκBα, IκBα, p-NF-κBp65, and NF-κBp65, and **(C)** nucleus NF-κBp65, Lamin B1, and cytosol NF-κBp65 were examined by Western blotting. ^*^ *P* < 0.05, ^**^ *P* < 0.01 versus the model group; ^##^ *P* < 0.01 versus the normal group.

Combining the results of the transcriptomic and molecular docking analyses, we speculated that HBOA might attenuate CALD by affecting the TLR4/NF-κB signaling pathway. As shown in Figures 6A, B, the expression levels of TLR4, MyD88, p-IKKα/β, p-IκBα, and p-NF-κBp65, measured by Western blot analysis, were significantly upregulated for the CALD model group, as compared with the normal group (*P* < 0.01). However, treatment with HBOA strikingly reversed these changes in a dose-dependent manner (*P* < 0.05). Furthermore, the data revealed that alcohol-induced expression of nuclear NF-κBp65 was significantly inhibited by HBOA, whereas the expression of cytosolic NF-κBp65, which was inhibited by alcohol, was clearly increased by HBOA in a dose-dependent manner (*P* < 0.01; Figure 6C). It is suggested that HBOA has a therapeutic effect on rats with CALD by inhibiting the TLR4/NF-κB signaling pathway, especially inhibiting NF-κBp65 translocation to the nucleus, thereby reducing the production of pro-inflammatory factors.

### 3.6 Effect of HBOA treatment on metabolites

A series of reactions during the metabolism of alcohol in the liver leads to disorders of liver metabolism; therefore, in this study, we used non-targeted metabolomics to examine the effects of HBOA on the metabolic profile of rats with CALD. The PCA plot revealed a relatively tight clustering among the QC samples, and Hotelling’s T2 Range revealed a high correlation coefficient. Furthermore, the total ion chromatograms (TICs) showed nearly the same shape. These data suggested that the conditions for metabolic analyses were stable and could be used for following assays (Supplementary Figures S1A–C).

**FIGURE 7:**
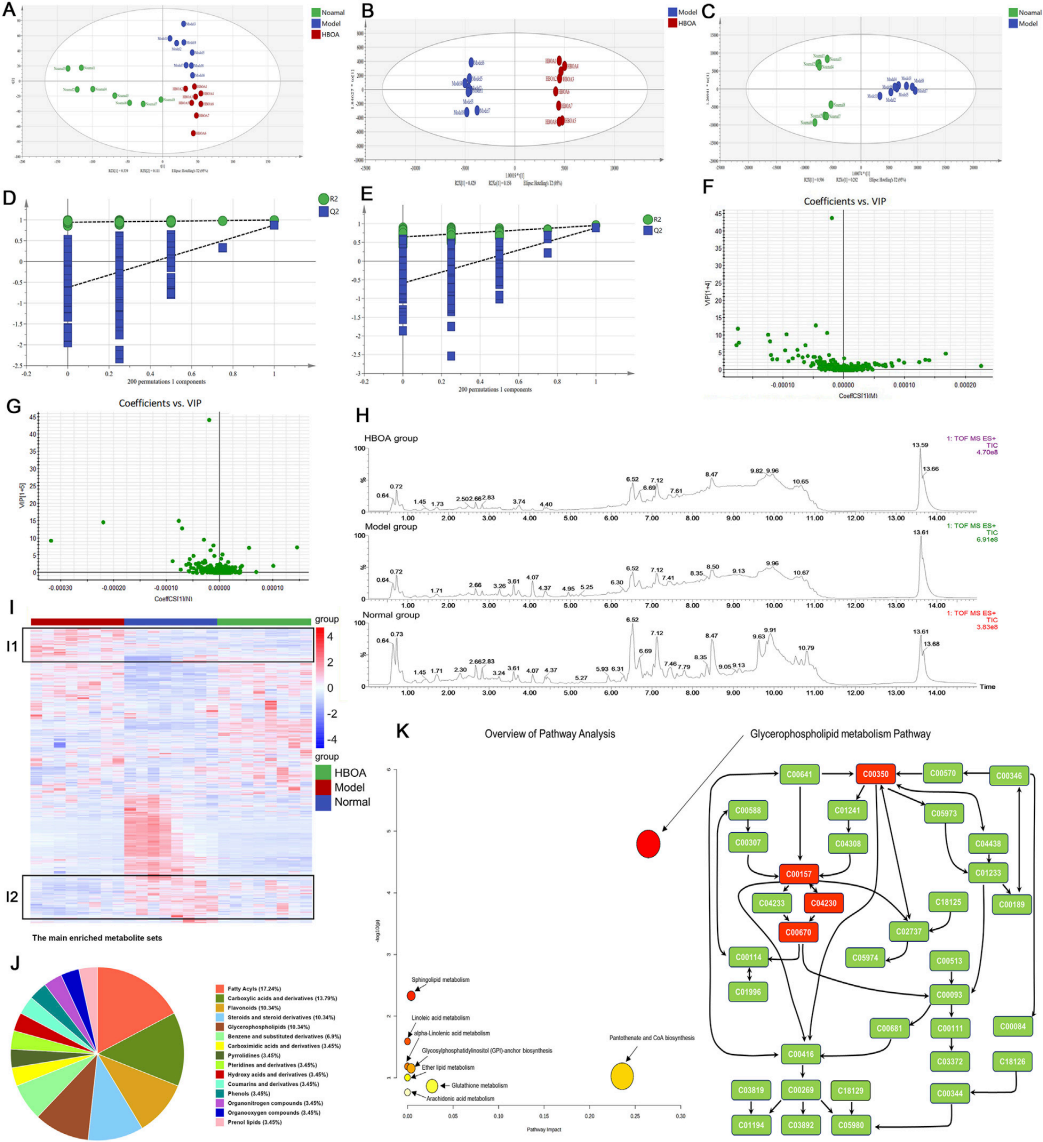
Effect of HBOA treatment on metabolites. **(A–C)** PCA and OPLS-DA analysis by SIMCA^®^. **(D)** 200 permutation Test of HBOA group VS. Model group. **(E)** 200 permutation Test of normal group VS. Model group. **(F)** VIP plot of HBOA group VS. Model group. **(G)** VIP plot of normal group VS. Model group. **(H)** Total Ion Flow Chart (TIC) **(I)** Heatmap analysis by R package; red represents high-abundance and blue represents low-abundance metabolites. **(J)** Chemical sorting enrichment analysis. **(K)** Analysis of HBOA-regulated metabolic pathways using MetaboAnalyst 5.0. Glycerophospholipid metabolic pathway.

The PCA diagram revealed a distinct distribution among the normal, model, and HBOA (100 mg/kg)-treated groups (Figure 7A). The OrthogonalPartialLeast Squares-DiscriminantAnalysis (OPLS-DA) plots also showed significant separation between the normal and model groups, and between the model and HBOA (100 mg/kg) groups (Figures 7B, C), which was further confirmed by the heatmap plot and TICs (Figures 7H, I). In addition, the permutation test indicated that the modeling was not over-fitted (Figures 7D, E). Discrepant compounds were searched via variable importance in the projection (VIP), and after filtering with the condition of VIP value ≥1 and *P* < 0.05, 78 metabolites differed between the model and HBOA (100 mg/kg) groups (Figures 7F, G). Chemical classification enrichment of metabolites revealed that these metabolites were mainly Fatty Acyls, Carboxylic acids and derivatives, Flavonoids, Steroids, steroid derivatives, and Glycerophospholipids (Figure 7J). The relevant metabolic pathways were then analyzed by MetaboAnalyst 5.0, and the results suggested that the metabolites that differed between the HBOA (100 mg/kg) and the model groups were mainly enriched in the glycerophospholipid metabolism pathway (Figure 7K). The results indicated that C00157 (Phosphatidylcholine), C00350 (phosphatidylethanolamine), C04230 (2-Lysophosphatidylcholine), and C00670 (glycerophosphocholine) were likely the targets of HBOA for regulating glycerophospholipid metabolism.

### 3.7 Integrative analysis of transcriptomics and metabolomics

**FIGURE 8:**
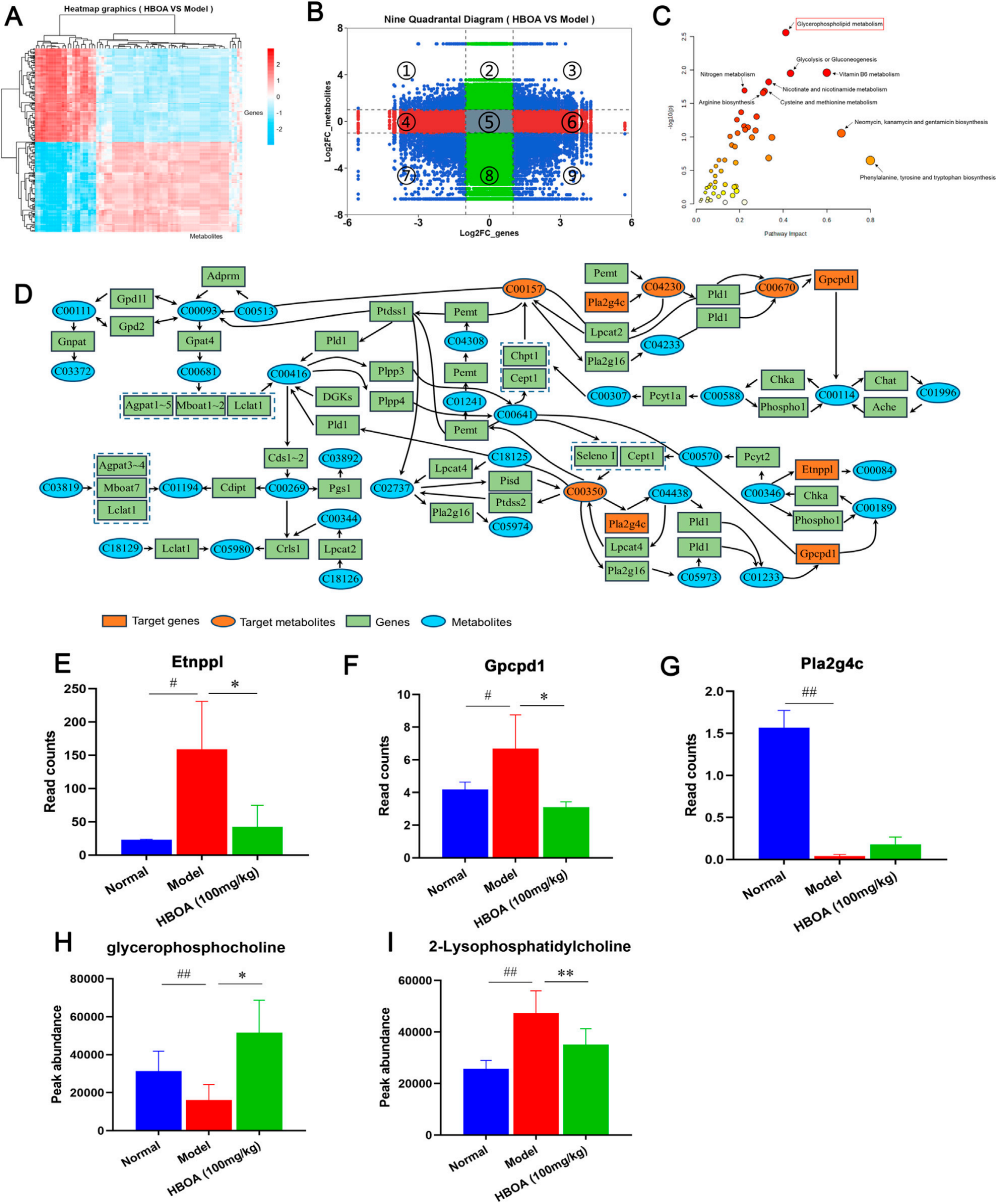
Integrated analysis of transcriptomics and metabolomics. **(A)** Correlation heat map. **(B)** 9-quadrant map. **(C)** Bubble plot of the Joint-Pathway analysis of differential metabolites and differential genes. **(D)** Integration of metabolite and gene relationships in the glycerophospholipid metabolic pathway. **(E–I)** The contents of Glycerophospholipid metabolism pathway-related metabolites and the expression levels of the target genes.

To further elucidate the relationship between the transcriptome and the metabolome, the correlation heatmap and 9-quadrant diagrams were drawn, which indicated that many genes might play direct or indirect regulatory roles in the alterations of the corresponding metabolites (Figures 8A, B). Therefore, we performed a Joint-Pathway analysis of differentially expressed genes and metabolites between the model and HBOA (100 mg/kg) groups, and found that HBOA exerted the greatest comprehensive effect on the glycerophospholipid metabolic pathway (Figure 8C). Further analysis of the glycerophospholipid metabolic pathway revealed that HBOA may affect the synthesis of phosphatidylcholine, phosphatidylethanolamine, 2-Lysophosphatidylcholine, and glycerophosphocholine by regulating the expression of *Pla2g4c*, *Gpcpdl*, and *Etnppl* genes, which ultimately regulate the glycerophospholipid metabolic pathway (Figure 8D). Ultimately, Read counts and Peak abundance of the above targets were statistically analyzed. As shown in Figure 8E–I, HBOA treatment significantly decreased *Etnppl*, *Gpcpd1*, and 2-Lysophosphatidylcholine expressions and increased glycerophosphocholine expression compared with the model group (*P* < 0.05). These results suggested that HBOA ameliorated CALD by inhibiting the glycerophospholipid metabolic pathway in rats.

## 4 Discussion

Liver disease, caused by alcohol abuse, is one of the leading causes of death worldwide (Seitz et al., 2018); the main features of the disease include steatosis, oxidative stress, abnormal lipid metabolism, and inflammation response due to alcohol-mediated changes in intestinal permeability (Dukić et al., 2023). In this study, we induced CALD animal models with various concentrations of alcohol and administered HBOA for treatment after successful modeling as determined by pathological examination during the modeling process. This was done to explore the therapeutic effect of HBOA on rats with CALD and determine its potential mechanisms. In reality, HBOA reduced damage in liver tissue caused by alcohol, such as congestion, necrosis, inflammatory cell infiltration, and steatosis. This was further confirmed by conducting tests to determine liver function and lipid levels. AST, ALT, and ALP are often used in clinical practice to determine the status of liver function as well as to diagnose liver disease (Huang et al., 2023; Kwo et al., 2017). Notably, we found that HBOA inhibits them. In addition, chronic alcohol intake is known to cause disturbances in lipid metabolism, affecting serum lipid levels in the body (Guo et al., 2022; Zhou et al., 2021). In this study, examination of serum lipid levels revealed that HBOA improved lipid metabolism. These results suggest that HBOA has a therapeutic effect on CALD in rats.

One mechanism underlying the development of CALD is overactivation of the innate immune response (Ilyas et al., 2019). Immune inflammation response is one of the main features of CALD (Rodina et al., 2021). Alcohol leads to the release of large amounts of intestinal-derived LPS into the liver through the portal circulation, which stimulates the immune system to produce a series of pro-inflammatory factors such as IL6, IL-1β, and TNF-α, causing infection and organ damage (Szabo and Bala, 2010; Zhao et al., 2014). The present study showed a significant reduction in pro-inflammatory factors and LPS levels in liver tissue after HBOA treatment. Redox reactions are important biochemical reactions that maintain the normal activities of bodily function. Excessive intake of alcohol leads to an increase in peroxides in the body, thus disrupting the body’s oxidative balance and inducing oxidative stress (Lu et al., 2021). MDA is considered to be one of the end products of lipid peroxidation, whereas SOD, GSH-Px, and GSH are important antioxidant enzymes that respond to the severity of oxidative stress in terms of their levels or activity within the body (Chen J. et al., 2023; Mai et al., 2022). In this study, HBOA inhibited the alcohol-induced increase in MDA levels and increased the activity of the antioxidant above enzymes. Therefore, HBOA inhibits the release of pro-inflammatory factors and oxidative stress in the liver of rats with CALD.

Alcohol metabolism in the body, either by itself or via its metabolites, disrupts metabolic homeostasis (Teschke, 2018). Transcriptomics indicated 334 differential genes between the HBOA-treated and model groups. GO analysis suggested that these differential genes are strongly associated with lipid homeostasis, localization, and transport. In addition, molecular signals from the cell exterior are transmitted through the cell membrane to trigger a series of enzymatic reactions within the cell that allow it to respond promptly to environmental changes. Examining metabolic and signaling pathways can reveal the mechanisms of disease or drugs. In this study, metabolic pathway and signaling pathway enrichment revealed that the differentially expressed genes were associated with glycerophospholipid and cholesterol metabolic pathways, as well as the MAPK, FoxO, AMPK, JAK-STAT, and NF-kappa B signaling pathways. By molecular docking, we found that HBOA exhibited the best binding activity with NF-κB and good binding activity with TLR4 (the pattern recognition protein of LPS), an upstream target of NF-κB. The transcription factor NF-κB activates inflammatory pathways and pro-inflammatory cytokines and plays an important role in the inflammatory response in alcoholic liver disease (Zhu et al., 2017). We believed that NF-κB had the best binding activity with HBOA and that the NF-κB signaling pathway might be one of the target pathways of HBOA in the treatment of CALD, and we verified this speculation through subsequent experiments.

The results of transcriptomic and molecular docking analyses suggested that the TLR4/NF-κB signaling pathway may be a target pathway for HBOA to attenuate the inflammatory response in rats with CALD. TLR4 is an upstream protein target of NF-κB as well as a pattern recognition receptor for LPS. Alcohol has been shown to break the intestinal barrier (Meroni et al., 2019), causing a large amount of LPS produced by intestinal bacteria to reach the liver through portal circulation (Rao, 2009). The TLR4 receptor is activated when the upstream signal reaches the cell membrane surface; MyD88 activation leads to IKKα/β activation; IκBα protein phosphorylation releases NF-κBp65 protein in the free state, which enters the nucleus to bind to the NF-κB promoter site of DNA, increasing the transcription of pro-inflammatory factors such as TNF-α, IL-6, and IL-1β, and resulting in inflammatory responses *in vivo* which eventually cause tissue damage (Endale et al., 2013; Funakoshi-Tago et al., 2016; Liu et al., 2020). In this study, Western blotting experiments showed that HBOA treatment decreased the expression of TLR4 and MyD88 and inhibited the phosphorylation of IKKα/β, IκBα, and NF-κBp65. Furthermore, following HBOA treatment, the transfer of NF-κBp65 to the nucleus was inhibited, suggesting that HBOA may counteract inflammation by inhibiting the TLR4/NF-κB signaling pathway.

The presence of metabolic risk factors is common in alcoholics (Åberg et al., 2020), with metabolic disorders leading to a variety of dysfunctions, including liver damage (Li et al., 2022). In this study, based on the findings of the transcriptomic analysis, we focused on whether the therapeutic effect of HBOA on CALD was associated with metabolic changes. We performed untargeted metabolomic analysis of liver tissue homogenates and found that 78 metabolites were altered in rats with CALD after HBOA treatment (*P* < 0.05, VIP >1). The results showed that these differential metabolites were mainly enriched in the glycerophospholipid metabolic pathway (*P* < 0.05, impact >0.1), which is consistent with the metabolic pathway enrichment analysis of differential genes in transcriptomics. Combined transcriptomics and metabolomics analysis suggested significant *Etnppl*, *Gpcpd1,* and *Pla2g4c* changes. In reality, *Etnppl*, a newly identified metabolic enzyme, catalyzes phosphoethanolamine to ammonia, inorganic phosphate, and acetaldehyde and is highly expressed in hepatic tissue (Wang et al., 2023). Moreover, overexpressing *Etnppl* may promote insulin resistance and generate reactive oxygen species (ROS) (Chen X. et al., 2023). Xiong et al. suggest that downregulation of *Etnppl* may inhibit glycerophospholipid metabolic pathways to ameliorate hepatic fibrosis (Xiong et al., 2022). In the present study, we found that the expression of *Etnppl* was significantly decreased in the HBOA (100 mg/kg) group, so we hypothesized that *Etnppl* may be one of the targets to improve CALD by affecting glycerophospholipid metabolism. The encoding product of *Gpcpd1* is EDI3, but we know little about its physiological functions (Glotzbach A et al., 2024). A study showed that the encoded product of the *Gpcpd1* gene plays a role in muscle ageing and age-associated glucose intolerance (Cikes et al., 2023). In addition, it has been shown that the encoded product of *Gpcpd1* can hydrolyze glycerophosphocholine to produce choline and G3P, which is the backbone molecule of all glycerophospholipids (Hishikawa et al., 2014; Stewart et al., 2012). Our study found that HBOA inhibited the expression of *Gpcpd1*, which was also indicated by the increased amount of glycerophosphocholine in the HBOA (100 mg/kg) group, and we therefore hypothesized that HBOA may inhibit the glycerophospholipid metabolic pathway by downregulating *Gpcpd1*. *Pla2g4c* mediates arachidonic acid production and activates lysophosphatidylcholine expression to trigger inflammatory signaling (Li et al., 2016; Stewart et al., 2002). Interestingly, Szymczak-Pajor I et al. found that 1,25(OH)2D3 treatment increased *Pla2g4c* expression in mast cells (Szymczak-Pajor et al., 2020). In addition, wu et al. found that *Pla2g4c* is lowly expressed in patients with homozygous familial hypercholesterolemia patients and suggested that it is involved in inflammatory signaling, but because of the presence of a protective negative feedback effect manifested as a decrease in the expression of *Pla2g4c* (Wu et al., 2021). Our study found that the expression of *Pla2g4c* was decreased in the model group. Still, the expression of 2-Lysophosphatidylcholine was increased in the model group, and we hypothesized that *Pla2g4c* participated in the inflammatory response and that the decrease in its expression might be related to protective negative feedback, but further studies are necessary to study this phenomenon. In summary, we hypothesized that HBOA may affect the expression of *Etnppl*, *Gpcpd1,* and *Pla2g4c* to improve glycerophospholipid metabolism.

## 5 Conclusion

**FIGURE 9:**
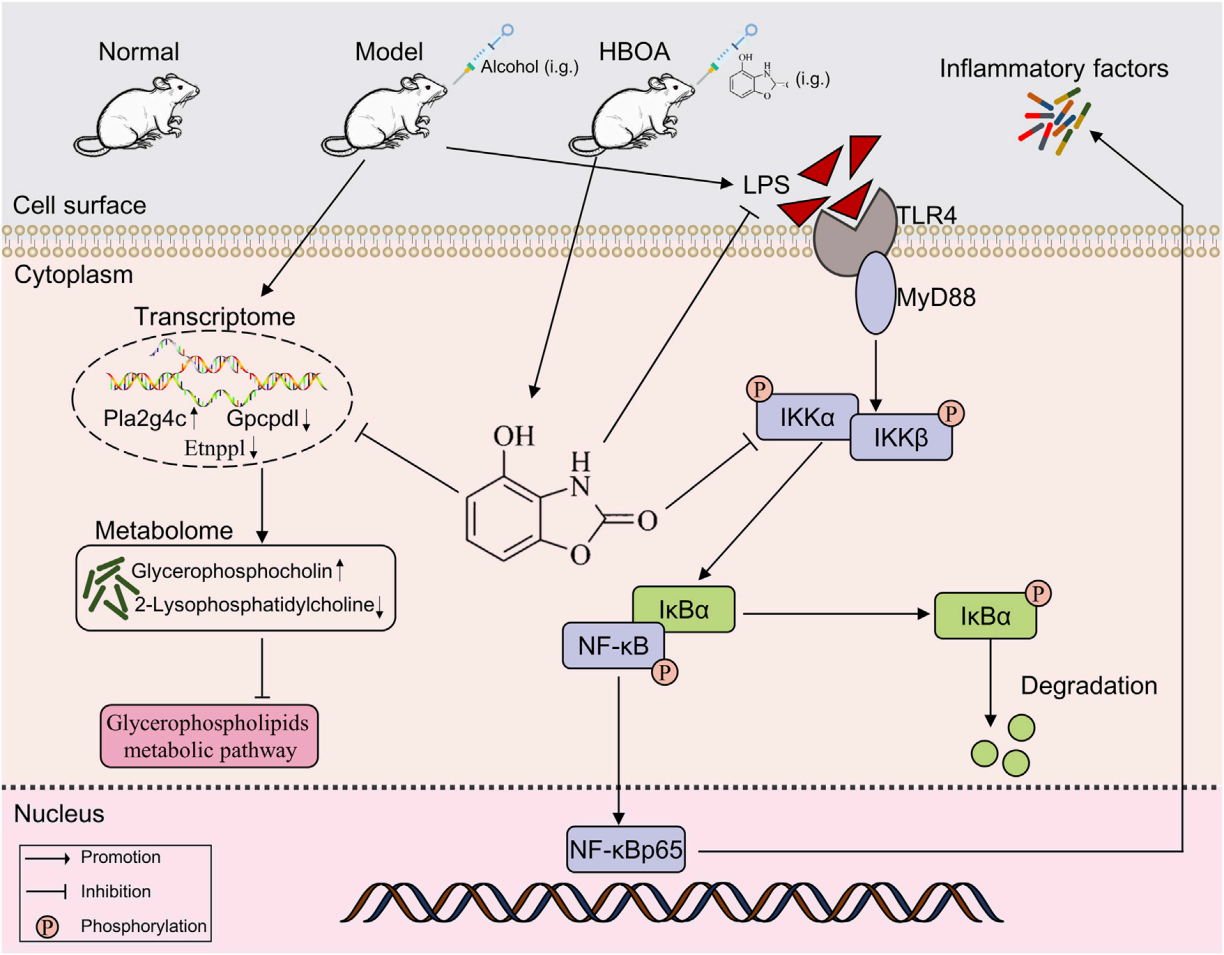
HBOA ameliorates CALD by inhibiting the TLR4/NF-κB signaling pathway, as well as by mediating glycerophospholipid metabolism.

In this study, it was shown for the first time that HBOA exerts a therapeutic effect on rats with CALD; further, the mechanism of action of HBOA was preliminarily elucidated. In summary, HBOA alleviates CALD, and its mechanism of action may be via the inhibition of the TLR4/NF-κB signaling pathway and glycerophospholipid metabolic pathway, which in turn plays an anti-inflammatory and regulatory role in metabolic homeostasis (Figure 9). In addition, we found for the first time that HBOA may affect glycerophospholipid metabolism by regulating the expression of *Etnppl*, *Gpcpd1* and *Pla2g4c* to improve CALD, and these genes may be a potential target for the treatment of CALD, but further studies are needed.

## Data Availability

The datasets presented in this study can be found in online repositories. The names of the repository/repositories and accession number(s) can be found below: https://www.ncbi.nlm.nih.gov/, PRJNA1145128.
