# Supplementary material for: Elucidating the role of 4-hydroxy-2(3H)-benzoxazolone in chronic alcoholic liver disease via transcriptomics and metabolomics
Source: Front Pharmacol. 2024 Sep 11;15:1447560. doi: 10.3389/fphar.2024.1447560 (PMC11422225; doi:10.3389/fphar.2024.1447560)
Supplement: Supplementary file 1 [file DataSheet1.pdf]

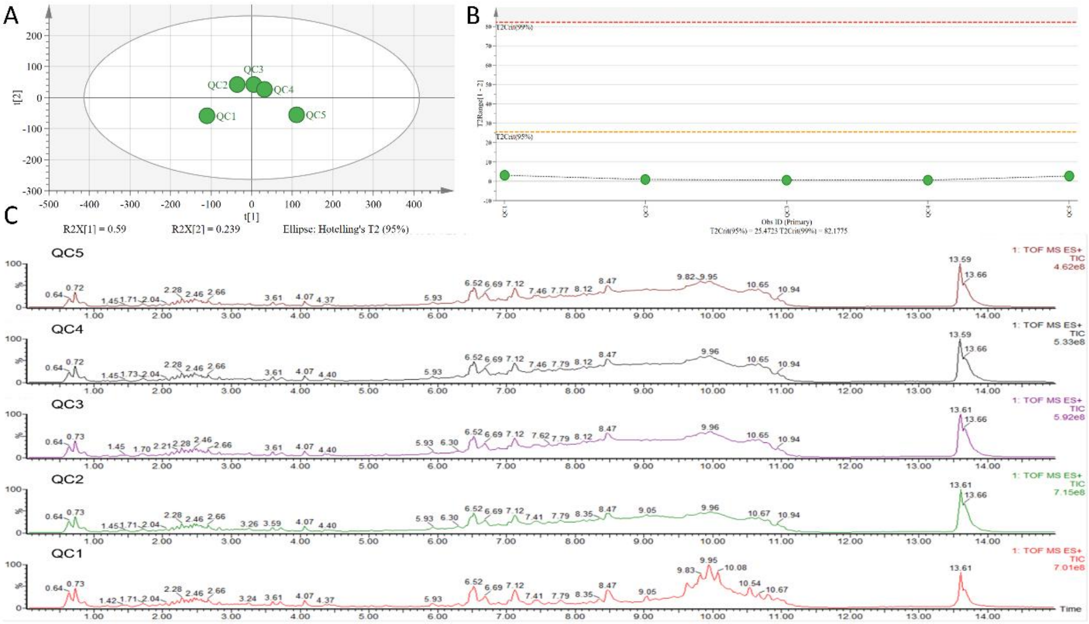

Figure S1. Quality control (QC) samples. (A) The PCA score map, (B) The Hotelling T2 map, (C) The Total Ion Flow Chart (TIC).
